# Varieties of Food—Their Chemical Composition and Nutritive Value

**Published:** 1869-06

**Authors:** 


### ARTICLE XI.

### *Varieties of Food—their Chemical Composition and Nutritive Value.*

*Animal Foods.*—First on the list of these is *milk*, a liquid which contains all the elements of food required by the very young, and is therefore regarded as the type or standard of food.

In some countries, as Switzerland, it is the chief diet of the peasantry; and everywhere, if easily obtained, it is largely consumed. 76 per cent. of the laboring classes of England make use of it; 83 per cent. take it as butter-milk, and 53 per cent. as skimmed-milk. In Wales, the average consumption of it by farm laborers is $4\frac{1}{4}$ pints per adult weekly—South Wales averaging only 3 pints, while in North Wales it is $7\frac{1}{2}$. In Scotland the consumption among the laboring classes is still larger, for it amounts to $6\frac{1}{4}$ pints per head weekly, and in Ireland it reaches $6\frac{3}{4}$ pints. Those who take

* From the Cantor Lectures, delivered before the Society of Arts, by Dr. Letherby, M. A.

least of it are the poor in-door operatives of London; the weavers of Spitalfield, for example, use only about 7·6 ozs. per head weekly, and those of Bethnal Green only a fraction above 1½ ozs. per head. When examined under the microscope, milk is found to consist of myriads of little globules of butter floating in a clear liquid. On standing for a few hours the oily particles rise to the surface and form a cream, the proportion of which is the test of quality. Cow's milk is heavier than water in the proportion of from 1,030 or 1,032 to 1,000. Asses' milk is the lightest, for its gravity is only about 1,019; then comes human milk, 1,020; and. lastly, goats' and ewes' milk, which is the heaviest of all, from 1,035 to 1,042.

The quality of milk varies with the breed of the cow, the nature of its food, and the time of milking, for afternoon milk is always richer than morning, and the last drawn than the first. Taking, however, the average of a large number of samples, it may be said that cow's milk contains 14 per cent. of solid matter, 4·1 of which are casein, 5·2 sugar, 3·9 butter, and 0·8 saline matter. The relations of nitrogenous to the carbonaceous is 1 to 2·2; but as fat is 2½ times more powerful than starch, the relation may be said to be as 1 to 3·6.

When milk is heated to the boiling temperature, the casein is coagulated to some extent; and if the milk has stood before it is heated, so that the cream may rise, the coagulum includes the cream, and makes the so-called Devonshire or clotted cream.

Acids also coagulate the casein, and produce a curd, as in the making of cheese and curds and whey.

*Cream* is rich in butter. It contains 34 per cent. of solid matter, 26·7 of which are butter, and its gravity is about 1,013.

*Skim-milk* is the milk from which the cream has been removed. It contains only about half as much butter as new milk, and its gravity is about 1,037. In all other respects it is similar to new milk.

*Buttermilk* is the residue of the milk of cream from which the butter has been removed by churning. It is still poorer in fat than skim-milk, containing, in fact, only about half as much. Unless it is very fresh, it is generally a little acid, and frequently the acidity has gone so far as to set the milk into a kind of jelly.

The whey of milk is the opalescent liquor from which the curd has been removed in making cheese. Although not highly nutritious, it still holds a little casein in solution, as well as the sugar and saline matter of the milk. It is rarely used as food by the poor, but is given to pigs. In Switzer land, however, it is considered to have medicinal virtues, especially for the cure of chronic disorders of the abdominal organs, and the treatment, which is somewhat fashionable, goes by the name of *cure de petit lait.* There is a popular notion that the whey of milk is sudorific, and hence we have our wine whey, cream of tartar whey, alum whey, tamarind whey, &c., when the milk has been curdled by these several substances.

*Cheese* is the coagulated product of milk, obtained by the addition of rennet or a little vinegar. When cream is coagulated it makes cream cheese, which will hardly bear keeping, but must be eaten fresh. It contains about half its weight of butter, and a fifth of its weight only of curd.

When cream is added to new milk, and the mixture is curdled, it forms very rich cheese, as double Gloucester and Stilton.

When new milk alone is used the cheese is less rich, but still of high quality, as Cheddar.

When an eighth or a tenth of the cream has been taken off, it produces the quality of cheese which is most sought after, as single Gloucester, Chester, American, &c.

And when all the cream has been removed, and the skim-milk is curdled, it forms the poor cheese of Holland, Friesland, Suffolk, Somersetshire, and South Wales.

At first every variety of cheese is soft and comparatively tasteless, but by keeping they undergo change, and develop their flavors, when they are said to be ripe.

Analyses of two of the most important of them show that they contain from 56 to 64 per cent. of solid matter, about half of which is curd. In skim-milk cheese the curd amounts to 44·8 per cent., and the fat to only 3·6; whereas, in Cheddar, the curd is only 28·4 per cent., and the fat 31·1. In nutritive power, therefore, especially in nitrogenous matter, cheese ranks high, and is a valuable article of diet; but there is a limit to its digestibility, and hence it cannot be taken in large quantity. Considering its price also, it is hardly so profitable as many other foods; although where good skim-milk cheese can be purchased at from 2½d. to 3d. per pound it forms, in small quantities at a time, a good adjunct to bread.

*Concluded in the July number.*

